# Comparative Study of Guanidine-, Acetamidine- and Urea-Based Chloroaluminate Electrolytes for an Aluminum Battery

**DOI:** 10.1021/acs.jpcc.3c05287

**Published:** 2023-09-15

**Authors:** Iwan Sumarlan, Anand Kunverji, Anthony J. Lucio, A. Robert Hillman, Karl S. Ryder

**Affiliations:** † Department of Chemistry, University of Mataram, Jl. Majapahit. No. 62, Mataram 83115, Lombok, Indonesia; ‡ Centre for Sustainable Materials Processing, School of Chemistry, University of Leicester, Leicester LE1 7RH, U.K.

## Abstract

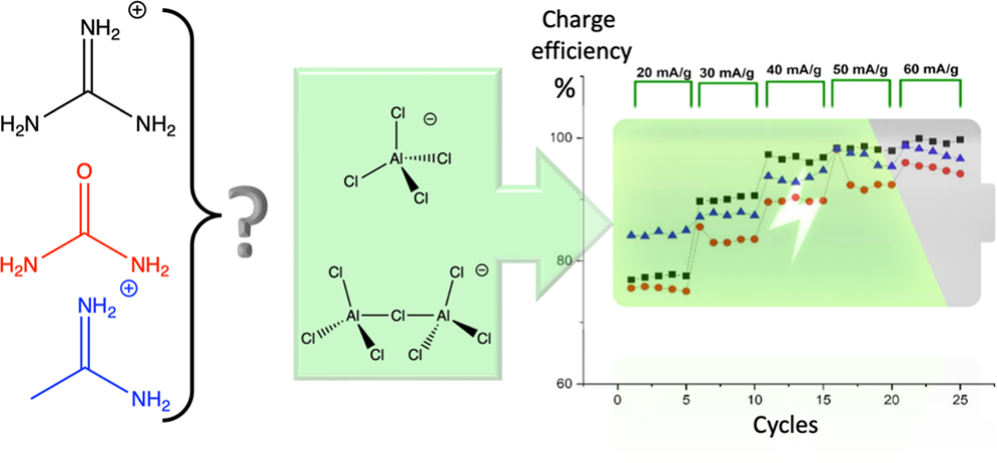

Aluminum-based batteries are a promising alternative to lithium-ion as they are considered to be low-cost and more friendly to the environment. In addition, aluminum is abundant and evenly distributed across the globe. Many studies and Al battery prototypes use imidazolium chloroaluminate electrolytes because of their good rheological and electrochemical performance. However, these electrolytes are very expensive, and so cost is a barrier to industrial scale-up. A urea-based electrolyte, AlCl_3_:Urea, has been proposed as an alternative, but its performance is relatively poor because of its high viscosity and low conductivity. This type of electrolyte has become known as an ionic liquid analogue (ILA). In this contribution, we proposed two Lewis base salt precursors, namely, guanidine hydrochloride and acetamidine hydrochloride, as alternatives to the urea-based ILA. We present the study of three ILAs, AlCl_3_:Guanidine, AlCl_3_:Acetamidine, and AlCl_3_:Urea, examining their rheology, electrochemistry, NMR spectra, and coin-cell performance. The room temperature viscosities of both AlCl_3_:Guanidine (52.9 cP) and AlCl_3_:Acetamidine (76.0 cP) were significantly lower than those of the urea-based liquid (240.9 cP), and their conductivities were correspondingly higher. Cyclic voltammetry (CV) and linear sweep voltammetry (LSV) showed that all three electrolytes exhibit reversible deposition/dissolution of Al, but LSV indicated that AlCl_3_:Guanidine and AlCl_3_:Acetamidine ILAs have superior anodic stability compared to the AlCl_3_:Urea electrolyte, as evidenced by anodic potential limits of +2.23 V for both AlCl_3_:Guanidine and AlCl_3_:Acetamidine and +2.12 V for AlCl_3_:Urea. Coin-cell tests showed that both AlCl_3_:Guanidine and AlCl_3_:Acetamidine ILA exhibit a higher Coulombic efficiency (98 and 97%, respectively) than the AlCl_3_:Urea electrolyte system, which has an efficiency of 88% after 100 cycles at 60 mA g^–1^. Overall, we show that AlCl_3_:Guanidine and AlCl_3_:Acetamidine have superior performance when compared to AlCl_3_:Urea, while maintaining low economic cost. We consider these to be valuable alternative materials for Al-based battery systems, especially for commercial production.

## 1 Introduction

Batteries are a key component of a sustainable and resilient energy system. Batteries contribute to the balance between the supply and demand on an electric grid by storing excess energy during periods of high production and releasing it at times of low production, as well as in the storage of energy for a vast range of portable modern technologies. Moreover, batteries can be used to provide backup power during power outages, which increases reliability and reduces disruptions in the power supply. The current market dominance and state-of-the-art lie in Li-ion technology because of its high energy and power densities and because of the advanced state of global manufacturing capability. However, there are serious concerns surrounding the long-term supply and sustainability of Li metal as well as cathode metals such as Ni, Mn, and Co that are currently driving the search for alternatives beyond lithium. Aluminum-based batteries (ABBs) have gained considerable attention in recent years and have become one of the promising alternatives to traditional lithium-ion batteries as they show high energy density and also both high volumetric capacity and gravimetric capacity (8040 mAh cm^–3^ and 2980 mAh g^–1^, respectively). Aluminum is also the most abundant metal element on the planet and is evenly distributed across the surface.

One of the key components of a battery is the electrolyte, which facilitates ion transfer between electrodes (cathode and anode). The rate of mass transfer in electrolytes is an important factor in determining the overall power density in operation. Finding an electrolyte suitable for aluminum batteries remains a difficult challenge. Aqueous ABB systems have been in operation as primary cells in military and other applications for some time, but recharging such cells is generally not possible because of hydrogen evolution and the formation of Al_2_O_3_ in the anode causing a relatively low standard electrode potential of aluminum (−1.662 V vs SHE).^1,2^ Academic focus has turned to chloroaluminate ionic liquids based on imidazolium, pyrrolidinium, and other organic salts, where reversible Al electrochemistry is facile; however, many of these organic cations are prohibitively expensive. More recently, many studies have focused on so-called deep eutectic solvent (DES) chloroaluminate liquids from combinations of AlCl_3_ and a Lewis base (LB) such as urea, acetamide, and others. These liquids are based on the reaction between an acid–base mixture comprising a Lewis acid (LA; i.e., AlCl_3_) and a Lewis base (LB; e.g., organic base or Cl^–^ containing salt). As these liquids often exhibit their best performance when formulated away from their eutectic composition, they have become known as ionic liquid analogues (ILAs). ILAs show other advantages, such as low vapor pressure and a wide electrochemical window, which are both favorable for highly reversible plating and dissolution efficiencies and are more suitable than aqueous systems for manufacturing ABBs.^3^ One such ILA is AlCl_3_:Urea.

The kinetic rate at which the active Al species in the IL or ILA can be reduced to Al metal (during the battery charge cycle) is highly dependent on the Al coordination sphere. For example, in the AlCl_4_^–^ ion, Al has a filled valence shell and so reduction of this species is both thermodynamically difficult and kinetically slow. On the other hand, partially satisfied species such as Al_2_Cl_7_^–^ are more easily reduced. Hence, control of Al speciation in ILAs is a key part of the design process and is dominated by the nature of the Lewis base. In classic chloroaluminate ionic liquids, for example, formulated from an imidazolium chloride and AlCl_3_, the ionic speciation in the electrolyte is dependent on the mole ratio, whether this be basic, neutral, or acidic. In the Lewis basic electrolyte, AlCl_4_^–^ and Cl^–^ coexist; in the Lewis neutral electrolyte, AlCl_4_^–^ is the dominant species (with very little free chloride); while in the Lewis acidic electrolyte, Al_2_Cl_7_^–^ is present.^4,5^ The reversible Al dissolution/deposition process is only possible in the presence of reducible Al_2_Cl_7_^–^. In chloroaluminate DES and ILAs, the Lewis base component is also capable of interacting with the Al center, and so the coordination processes and diversity of species are much more complex. In the case of the AlCl_3_:Urea ILA, the *O* lone pairs of the urea are believed to coordinate with the Al center.^6^ Aluminum is, however, highly oxophilic, and so the urea ligand may be a poor leaving group during electroreduction. This is likely to lead to slow reduction kinetics. A large number of recent studies have focused on urea-based ILA electrolytes^7−15^ as an alternative to 1-ethyl-3-methylimidazolium chloride (EMIM-Cl) in order to provide a more cost-effective solution for ABB systems. Recently, this topic was reviewed.^16^ In this context, the urea ILA makes a significant contribution and exhibits a reasonably good battery performance; it is a strong candidate for the next generation of ABBs. However, urea-based chloroaluminate ILAs have very low conductivity and very high viscosity and so are not ideal for the ABB system to function as intended. Such an electrolyte would be suitable only for applications of low operational power density. Hence, careful and informed selection of the LB component may offer control of both the liquid rheology and the energetics/kinetics of electrochemical reduction.

In our studies, we sought to both understand the chemistry and speciation of these chloroalumiate ILAs and also improve the rheological and electrochemical performance of the urea-based systems. At the same time, we sought to find liquid components that are economically viable for scale-up and which will cost less than existing electrolytes (such as imidazolium and pyrrolidinium salts). In this paper, we present a comparative study of three ILA electrolytes. These are formulated from stoichiometric combinations of AlCl_3_ with guanidine hydrochloride, acetamidine hydrochloride, and urea. These are currently more expensive than urea but much less expensive than imidazolium or pyrrolidinium salts.

Both guanidine and acetamidine salts possess the amidine functional group, which has only nitrogen atoms as Lewis base donors. These are likely to be softer Lewis bases than the oxygen of urea and so more facile ligands to the chloroaluminate aluminum center. The guanidine salt is unique in that the guanidinium cation has 3-fold symmetry so that all three *N*-atoms are equivalent. Our preliminary studies indicated that the properties of this ILA are favorable.^17^ The aim of this study was to understand the relation between the molecular structure of the LB component of the ILA and to find better alternatives to the urea-based electrolyte. To this end, we examined the rheological properties and electrochemical characteristics of the three liquids and performed ^1^H and ^27^Al NMR spectroscopies in order to gain some chemical insights into the speciation in the ILAs. Finally, we fabricated and tested coin-cell prototypes using the ILA electrolytes. To the best of our knowledge, this is the first paper to describe such a comparative study of these ILAs.

## 2 Experimental Section

### 2.1 Chemicals

The solid Lewis base (LB) salts acetamidine hydrochloride (≥98%, Acros-Organics), guanidine hydrochloride (99.99%, Sigma-Aldrich), and urea (ReagentPlus, ≥99.5%, pellets, Sigma-Aldrich) were used as received. The solid Lewis acid (LA) salt aluminum chloride (AlCl_3_; anhydrous, granular, 99%, Alfa Aesar) was used as received. Pyrolytic graphite (PG) produced by Panasonic, a 0.5 mm Pt disk (WE), a Pt flag electrode (CE), and the quasi-reference electrode (QRE) consisting of a straight, bare 2.0 mm diameter aluminum wire (99.9998%, metals basis, Alfa Aesar) were used. All materials were dried in vacuum at 60 °C for 24 h before being transferred into an Argon-filled glovebox.

### 2.2 ILA Preparation

AlCl_3_:Guanidine, ACl_3_: Acetamidine, and AlCl_3_:Urea were prepared with ratios of 2.0:1, 1.5:1, and 1.5:1, respectively, where inside the glovebox the concentration of H_2_O and O_2_ is less than 0.1 ppm. For AlCl_3_:Urea, AlCl_3_ was added slowly into urea at room temperature of 21 °C, producing a transparent light-yellow color. For both AlCl_3_:Guanidine and AlCl_3_:Acetamidine, AlCl_3_ was added into LB at 70 °C, producing transparent light-yellow and dark-purple colors, respectively. All three ILAs were stirred for at least 24 h and left at room temperature inside the glovebox before use.

### 2.3 Conductivity and Viscosity Measurements

The viscosity measurement was conducted at 25 °C by obtaining the resistance of the electrolyte using a Quartz Crystal Microbalance (QCM 922A). The electrode was a 9.00 MHz (±30 kHz) AT-cut quartz crystal resonator (Seiko) with Pt-coated electrolyte-facing and air-facing sides. The conductivity was measured using a conductivity probe (METTLER TOLEDO) and meter (Inlab 70 Personal Conductivity Sensor; METTLER TOLEDO). Before the measurement of the electrolytes, the conductivity meter was tested using standard electrolytes (12.88 mS cm^–1^; METTLER TOLEDO). The conductivity was measured at various temperatures in the range of 25–80 °C to obtain the activation energy of each electrolyte.^18^

### 2.4 NMR Measurement

NMR spectra were acquired using a Bruker AV500 spectrometer at ambient temperature. A 1M aqueous solution of Al(NO_3_)_3_ × 9H_2_O was used as a reference for ^27^Al. The reference solution was placed in a sealed glass insert placed inside the NMR tube.

### 2.5 Battery Preparation

The battery was assembled using a standard configuration coin-cell CR2032 (Cambridge Energy Ltd.). The coin cell consists of (1) a bottom cap (positive end), (2) a graphite-based cathode (PG) with a diameter of 14 mm, (3) a glass fiber filter paper separator (16 mm), (4) an Al sheet anode material with a diameter of 16 mm, (5) a 0.5 mm stainless steel (SS) spacer, (6) a 1 mm SS spacer, (7) an SS spring, and (8) an SS top cap (negative end). All stainless steel (SS) components are 316 grade. The Al foil and pyrolytic graphite were specially treated before they were assembled into the cell. To remove surface impurities, the Al foil was cleaned ultrasonically by washing it with ethanol for 10 min. The 14 mm PG was cleaned ultrasonically using deionized water for 10 min. Afterward, both materials were rinsed with ultrapure water and vacuum-dried.

### 2.6 Electrochemical Testing

Battery testing was conducted using a multichannel electrochemical analyzer (IVIUMnSTAT) with a cutoff voltage between 0.01 and 2.45 V (vs Al^(III)^/Al). The battery was set at open-circuit potentials (OCPs) for 2 h before the start of charge/discharge. Both cyclic voltammetry (CV) and linear sweep voltammetry (LSV) were conducted using a three-electrode configuration [0.5 mm Pt disk (WE), flag Pt electrode (CE), and the quasi-reference electrode (QRE)] in the potential window of −0.1 vs 1.0 V for CV and 0.0 vs 2.5 V for LSV with a scan rate of 10 mV s^–1^ for both.

## 3 Results and Discussion

**Figure 1:**
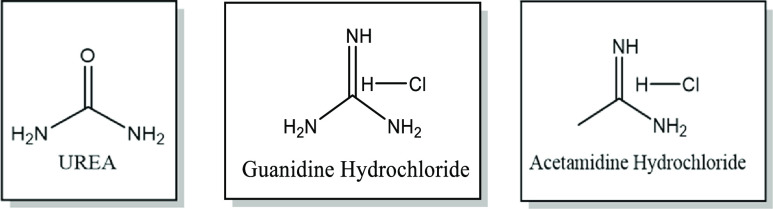
Chemical structure of Lewis bases: urea, guanidine hydrochloride, and acetamidine hydrochloride.

The three ILAs based on guanidine hydrochloride (AlCl_3_:Guanidine), acetamidine hydrochloride (AlCl_3_:Acetamidine), and urea (AlCl_3_:Urea) were synthesized by mixing the Lewis base with AlCl_3_ in an argon-filled glovebox with ratios of 2.0:1, 1.5:1, and 1.5:1, respectively. Figure 1 shows the chemical structures of the three Lewis bases. While there is a narrow range of possible compositions for each of these ILAs, these individual compositions were chosen for this study because in each case, they provided the liquid with the maximum value of conductivity (corresponding minimum values of viscosity) and best rheological stability at the ambient operational temperature. At room temperature, the ILAs were transparent light yellow in color for AlCl_3_:Guanidine and AlCl_3_:Urea and dark purple in color for AlCl_3_:Acetamidine (Figure S1). We tried to synthesize AlCl_3_:Guanidine with ratios of 1.50:1 and 1.75:1, but we found that neither of these compositions was stable and that a solid precipitate formed with time. Therefore, these two ratios cannot be used to achieve good performance in a battery.

### 3.1 Rheological Properties

Viscosity and conductivity are two parameters that play the most significant role with regard to the electrolyte in a battery system. These two parameters describe the mass and charge transport in the liquids, which directly influence the power and limiting current available from a cell. The viscosity and conductivity of the three ILA systems are presented in Table 1. Here, the viscosities of the three ILAs were measured according to eq 1 from the admittance spectrum of a quartz crystal acoustic resonator (QCM) submerged in the electrolyte.^17^ This quantifies the relationship between the QCM frequency (ω = 2π*f*), dynamic viscosity (η) expressed in units of grams per centimeter per second (g cm^–1^ s^–1^), and density (ρ) expressed in units of grams per cubic centimeter (g cm^–3^). As a calibration reference, water can be used to estimate the viscosity of ILA electrolytes. By dividing the equation for ILA by that for water, we are able to calculate ILA with η(ILA). In this method, ρ(ILA) = 1.3 g cm^–3^, η(water) = 0.01 g (cm^–1^ s^–1^), and ρ(water) = 1 (g cm^–3^).
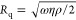
1

**Table 1:** Viscosity and Conductivity of the Three ILAs Measured at 25 °C^a^

ILA	viscosity (cP)	conductivity (mS cm^–1^)
AlCl_3_:guanidine (2.0:1)	52.9	9.33
AlCl_3_:acetamidine (1.5:1)	76.0	7.14
AlCl_3_:urea (1.5:1)	240.9	1.06

a The liquid composition (AlCl_3_:base ratio) is given in parentheses.

This method of determining viscosity is chosen here because it facilitates rapid (<1 s), reliable, and accurate measurements on small volumes (<5 cm^3^) of liquid in an *in situ* environment. We recently described this method in detail.^18^ In these circumstances, we determined viscosities of 0.529 g cm^–1^ s^–1^ for AlCl_3_:Guanidine (2.0:1), 2.41 (g cm^–1^ s^–1^) for AlCl_3_:Urea (1.5:1), and 0.760 (g cm^–1^ s^–1^) for AlCl_3_:Acetamidine (1.5:1) at 25 °C. For convenience, Table 1 shows the viscosity values converted to centipoise (cP).

The viscosity values found in this study are comparable to those reported in previous studies for the AlCl_3_:Urea (218–240 cP),^12,17,,21^ AlCl_3_:Guanidine (51 cP),^17^ and AlCl_3_:Acetamidine (30–70 cP) electrolytes.^18^

From the above data, among these three ILAs, we can conclude that the AlCl_3_:Guanidine electrolyte has the lowest internal resistance, resulting in a lower viscosity than those of AlCl_3_:Acetamidine and AlCl_3_:Urea. According to Yang et al.^22^ and Yu et al.,^23^ the internal resistance of the liquids is primarily determined by the interaction between cations and anions of ILAs, including hydrogen bonds, electrostatic interactions, and van der Waals interactions. Liu et al.^24^ concluded that AlCl_3_:Urea exhibits the highest viscosity as a result of increased LB bonding between urea and the Al coordination site. In the case of urea, there are Lewis basic lone pairs on both the oxygen and nitrogen atoms, but modeling indicates that the *O*-coordination is preferred.^6^ This is perhaps intuitive, given the highly oxophilic nature of aluminum. In the case of acetamidine and guanidine, only coordination through the *N*-atoms is possible, and these molecules are likely to be progressively (respectively) less Lewis basic than urea. In addition, while urea is a neutral Lewis base, both the guanidinium and acetamidinium species also carry positive charges; this is also likely to make them weaker bases. Hence, the observed trend in viscosity may be explained by the more facile exchange of the weaker ligand to the Al center. Furthermore, the viscosity of the urea liquid is known to considerably increase with an increase in the molar ratio of AlCl_3_:Urea (more acidic), whereas in this system, [AlCl_3_Ln] becomes more dominant in the liquids, resulting in increased viscosity. This also means that in the AlCl_3_:Urea system, there is less free volume to provide a “hole” that enables ion mobility^25^ compared to AlCl_3_:Guanidine and AlCl_3_:Acetamidine systems.

The corresponding conductivity values were obtained for the three ILAs and these followed an inverse trend with respect to viscosity. This is expected because the ionic mobility is naturally high in the low-viscosity medium. As a consequence of having the lowest viscosity among the three ILAs, the AlCl_3_:Guanidine system has the highest value of conductivity. In this study, we found that the conductivities of the three systems are 9.33, 7.14, and 1.06 mS cm^–1^ for AlCl_3_:Guanidine, AlCl_3_:Acetamidine, and AlCl_3_:Urea, respectively (Table 1). To observe the effect of temperature on the conductivity, the three ILAs were tested in a range of temperatures from 25 to 80 °C, and subsequently, the activation energy for viscous flow can be obtained from the measurement following the Arrhenius plot. The Arrhenius equation is expressed in eq 2.
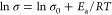
2where σ is the conductivity, σ_o_ is the constant related to the frequency factor, *E*_a_ is the activation energy, *R* is the gas constant (8.3145 J mol^–1^ K^–1^), and *T* is the absolute temperature in Kelvin.

**Figure 2:**
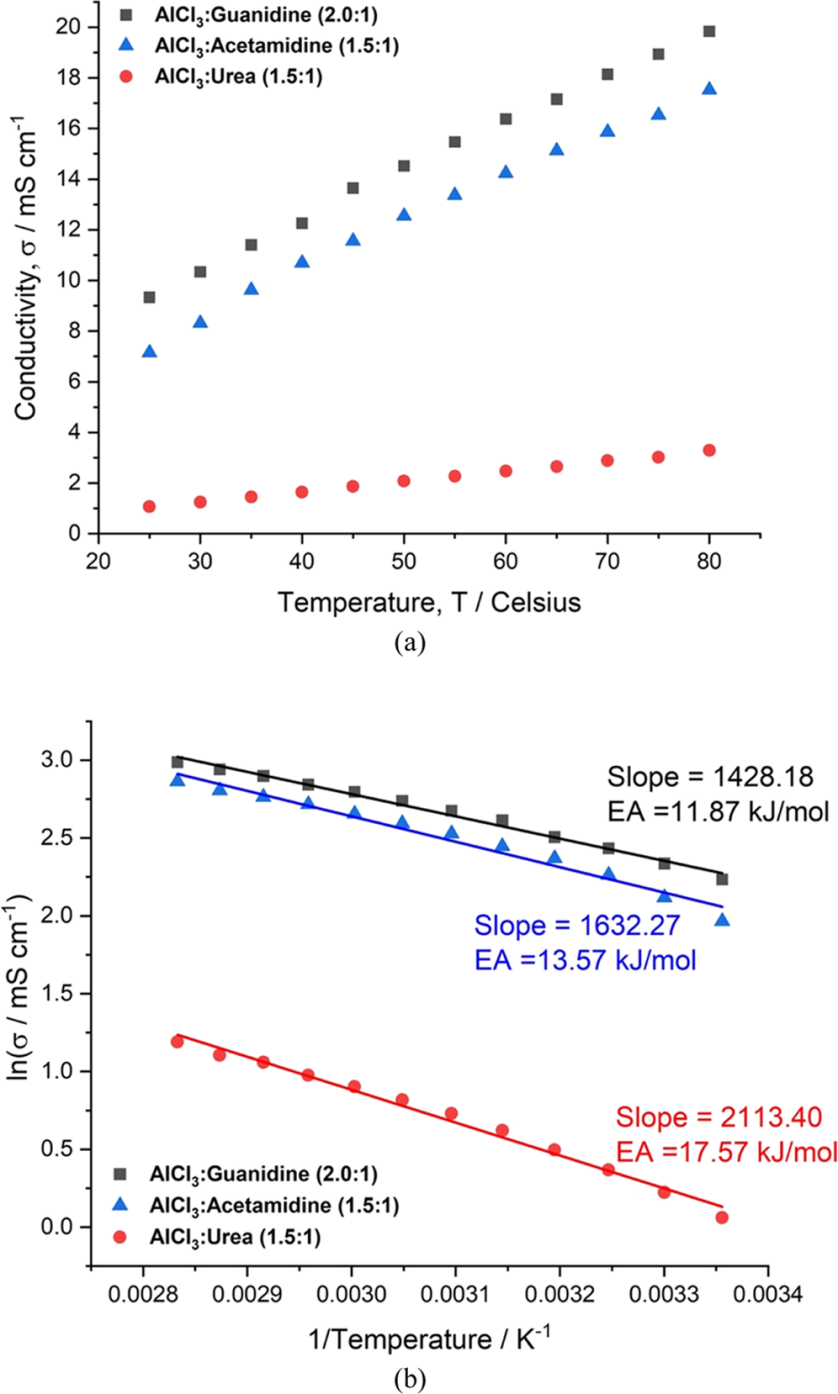
(a) Conductivity of the three ILAs as a function of temperature. (b) Arrhenius plot.

As expected, it is seen that an increase in the temperature leads to an increase in the conductivity of the ILAs (Figure 2a). In this case, this is not only due to the thermally activated mobility of the individual ions, but it also reflects the activity (concentration) of ionic species due to the temperature-dependent position of the various speciation equilibria. As a result, this suggests that an increase in temperature could significantly reduce the internal resistance of ILAs to flow and improve the charge transport as well. The charge transport in ILAs is governed by the statistical hole mobility at low temperatures, and it increases with temperature. As a consequence, it is more likely that ionic movement can occur into a void of appropriate dimensions, resulting in higher conductivity.^26,27^ To determine the activation energy, the Arrhenius equation above is employed, where the gradient of the plot is equal to *E*_a_/*R* (Figure 2b). It was found that the activation energy of the three ILAs are 11.87, 13.57, and 17.57 kJ mol^–1^ for AlCl_3_:Guanidine, AlCl_3_:Acetamidine, and AlCl_3_:Urea, respectively. The activation energy is defined as the amount of energy required for ions to move, and this therefore confirms that the ion mobility and charge transport in AlCl_3_:Guanidine are considerably higher than those in the remaining two systems.

### 3.2 NMR Spectroscopy (^1^H and ^27^Al) of the ILA Electrolytes

It is clear that the complexation of the metal in the ILA is an important topic as speciation affects redox potentials, solubilities, mobilities, and electroreduction kinetics. Recent reviews of speciation analysis techniques for chloroaluminate have been published. There are a number of parameters related to speciation that determine whether electrodeposition is possible in the manner or form desired. Nuclear magnetic resonance (NMR) spectroscopy is a well-known established method to probe the speciation in chloroaluminate, as shown by numerous previous studies. In the acidic ILAs, some Al species are present in the form of AlCl_4_^–^, Al_2_Cl_7_^–^, and AlCl_2_L*_n_^z^* (where L is the Lewis base ligand, and the overall charge, *z*, depends on the charge of L), and these species can be identified using ^27^Al NMR spectroscopy. In the liquids studied here, the LB components also contain hydrogen atoms and so ^1^H NMR spectroscopy is also useful.

**Figure 3:**
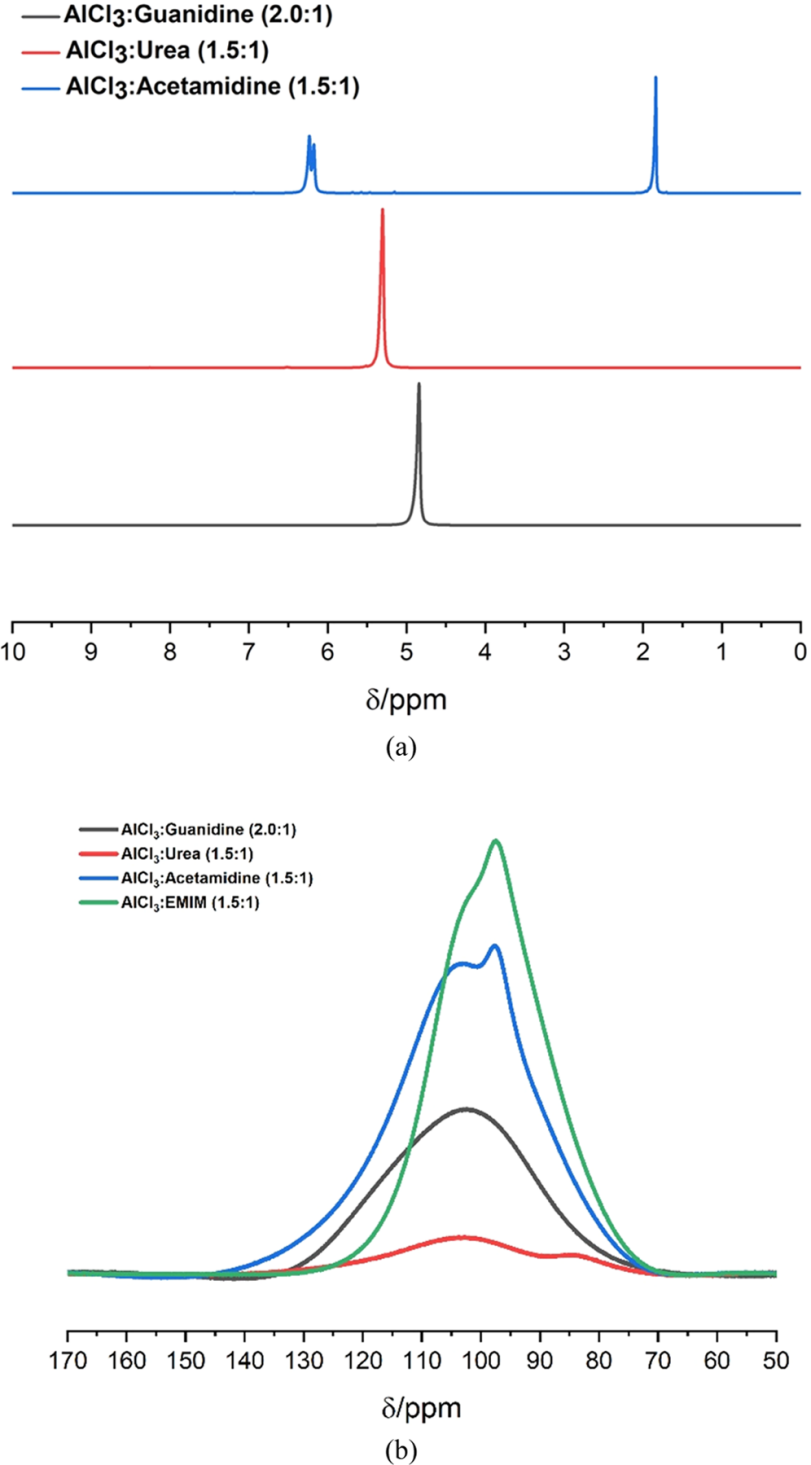
(a) ^1^H NMR of the three ILAs. (b) ^27^Al NMR for the three ILAs.

The ^1^H NMR spectra obtained for AlCl_3_:Guanidine, AlCl_3_:Acetamidine, and AlCl_3_:Urea are shown in Figure 3a. The ^1^H NMR spectra of the AlCl_3_:Guanidine and AlCl_3_:Urea ILAs showed a single peak at δ = 4.88 and 5.48 ppm, respectively. These represent the NH protons of each species and show a single environment. The ^1^H NMR spectra obtained from the AlCl_3_:Acetamidine ILA show a similar signal at δ = 6.30 ppm, which we also assign to the NH protons; however, in this case, we can see that the signal is split into two. In addition, for the AlCl_3_:Acetamidine ILA, there is a signal at δ = 1.84 ppm, which we assign to the –CH_3_ protons of the acetamidinium cation. The signal splitting in the –NH region for the acetamidine electrolyte is not caused by the spin–spin coupling to the protons of the –CH_3_ group because no reciprocal coupling is observed in the signal at δ = 1.84 ppm. Hence, it is likely that this splitting is caused either by the restricted rotation of the acetamidine ligand as it is bound to the Al site or by a slow exchange of the bound and unbound acetamidinium ligands. In comparison to typical organic solvents, the ^1^H chemical shifts for all three amides were within the expected range for all three groups. For the AlCl_3_:Urea system, the chemical shift (δ = 5.48 ppm) occurs in the same region reported by Malik et al.^26^ These results suggest that there is no bulk chemical reaction between AlCl_3_ and guanidine, acetamidine, or urea or degradation of the LB component during formulation. In other studies, the hydrolysis of chloroaluminates was investigated by ^1^H NMR, and Ferrara et al.^27^ observed a signal at around δ = 0.0 ppm in an AlCl_3_-[EMIM]Cl liquid in the presence of water. We observed no such signal here for any of the ILAs, and so we conclude that there is no evidence of hydrolysis of the ILAs during synthesis or as a result of handling.

The ^27^Al NMR spectra for the three neat liquids are shown in Figure 3b along with the ^27^Al NMR spectrum of the commercial AlCl_3_-[EMIM]Cl liquid (2:1) as a reference for comparison. These spectra show wide broad peaks for the four acidic ILAs between 70 and 150 ppm, which are characteristics of aluminum species in chloroaluminate. This is typical of ^27^Al NMR in such liquids because the nuclear spin, *I*, of the ^27^Al nucleus is *I* = 5/2, and therefore, the Al nucleus is quadripolar, which results in signal broadening. The intensities of the signals vary markedly between samples, but this may be due to a combination of experimental factors including line-broadening effects. It is tempting to attribute the different intensities to differences in the concentration of Al species in each electrolyte; however, the total concentration of Al species in each electrolyte is as follows: 7.8 mol dm^–3^ for AlCl_3_:Urea, 7.5 mol dm^–3^ for AlCl_3_:Guanidine, and 6.9 mol dm^–3^ for AlCl_3_:Acetamidine.^17^ Hence, the urea electrolyte has the least intense ^27^Al NMR signal despite having the highest concentration. However, the urea electrolyte has the highest viscosity, and this is known to have a broadening effect.

In the observed region, two overlapping peaks are seen in the spectra for the AlCl_3_:Acetamidine, AlCl_3_:EMIM, and AlCl_3_:Urea liquids, and one broad peak is seen for the AlCl_3_:Guanidine ILA. The peaks at δ = 98 ppm and δ = 103 ppm in AlCl_3_:Acetamidine and AlCl_3_:EMIM liquids were assigned to the aluminum species Al_2_Cl_7_^–^ and AlCl_4_^–^, respectively^12,24,25,27^ Here, the differing line widths are associated with the symmetry and mobility of the species. In addition, according to a previous study,^28^ broad peaks close to δ = 103 ppm are also assigned to species resulting from the replacement of chlorine with neutral or oxygen-based ligands (L), for example, [AlCl_3_L] and [AlCl_2_L_2_]^+^.

In the AlCl_3_:Urea system, the broad peak close to δ = 102 ppm may also be assigned to a mixed species environment including urea as a ligand, e.g., [AlCl_2_(urea)_2_]^+^ or [AlCl_3_(urea)]^6^, in addition to AlCl_4_^–^ and Al_2_Cl_7_^–^. The spectrum of the urea liquid shows an additional broad peak at δ = 88. Previous studies indicated that this peak can be associated with mixed ligand urea species, including [AlCl_3_(urea)], [AlCl_2_(urea)_2_]^+^. This is characteristic of the acidic AlCl_3_:Urea system, in line with previous studies.^11,12,26,29^ The Al species and associated chemical shifts identified from these literature studies are presented in Table 2.

**Table 2:** Likely Speciation of Al Ions Present in Chloroaluminate ILAs Identified Based on ^27^Al NMR Studies Present in the Literature^a^

Al species^–^	chemical shift, δ (ppm)	literature source (ref)
AlCl_4_^–^	103	(12), ^26^, ^27^
Al_2_Cl_7_^–^	97	(26), ^27^
AlCl_3_L	90, 89	(12), ^26^
AlCl_2_L_2_^+^	74, 75	(12), ^26^

a L = urea.

The very broad peak at δ = 103 ppm in the AlCl_3_:Guanidine spectrum may be assigned to a combination of species with chloride and guanidinium mixed coordination in addition to AlCl_4_^–^ and Al_2_Cl_7_^–^, which are also probably present. This single broad peak may be a result of a qualitatively fast, on the ^27^Al NMR time scale, exchange between the coordination environments of the species.^30^ This suggests that the ligand binding/exchange may be relatively facile, which is beneficial for electrochemical reduction.

### 3.3 Voltammetry of the ILAs

**Figure 4:**
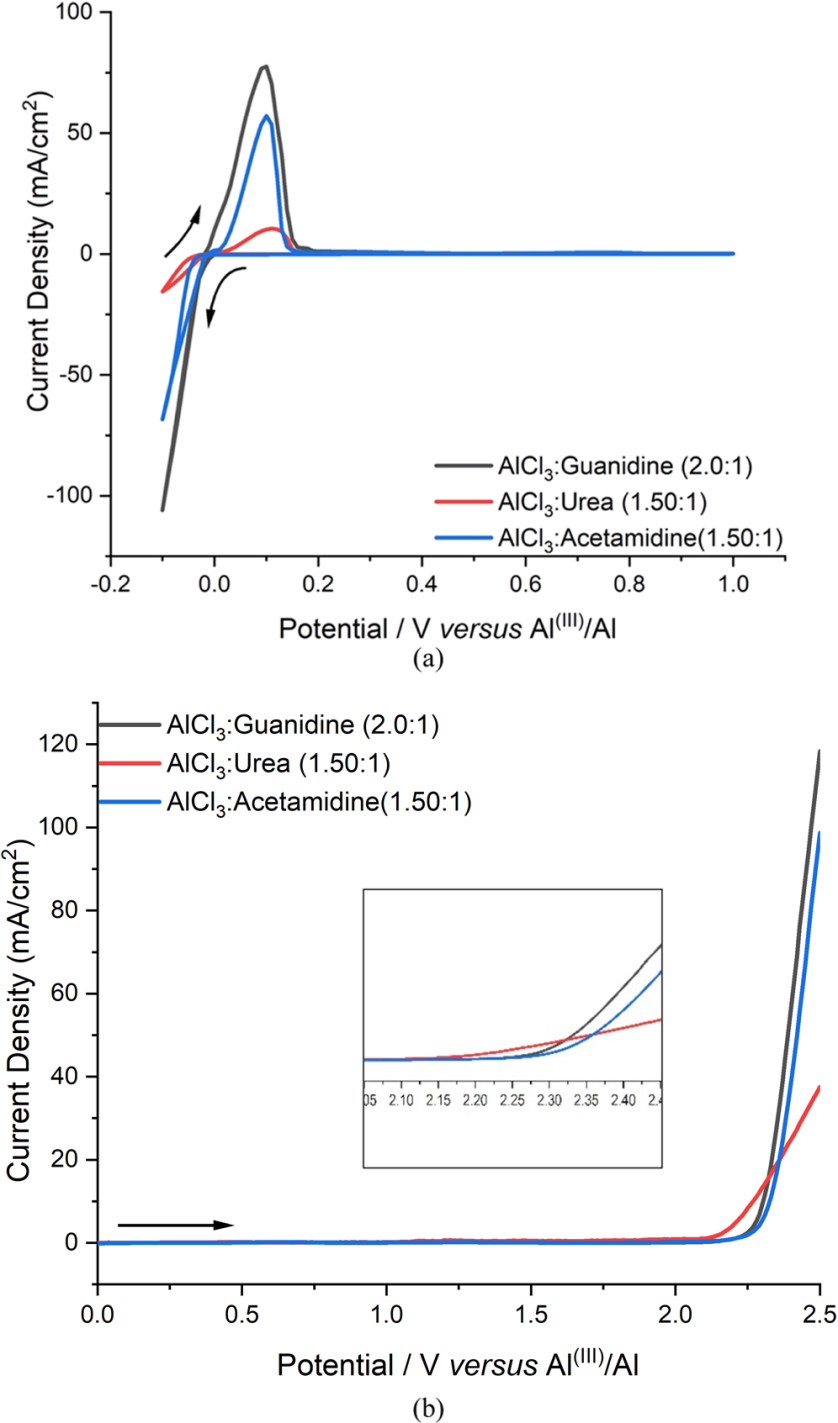
(a) Cyclic voltammetry (CV) and (b) anodic linear sweep voltammetry (LSV) of the three ILAs; inset shows the expanded region of the potential scale. Both CV and LSV recorded against an Al wire reference electrode at a potential scan rate of 10 mV s^–1^.

Cyclic voltammetry (CV) and linear sweep voltammetry (LSV) were performed to investigate the electrochemical stabilities of the three ILAs. In order to determine whether the CV cycle number affected the results, the five cycles were examined. Once five full cycles were completed, there were no discernible differences in the CV response (Figure S2). Figure 4a shows an experimental CV depicting the response associated with aluminum deposition and dissolution from the three ILA formulations on a Pt electrode at 10 mV s^–1^ in the potential range of −0.1–1.0 V (versus Al^(III)^/Al). The cathodic potential limit of the voltammetric scan was carefully chosen to minimize any contribution from electrolyte degradation. This is generally evidenced by a high current efficiency. This aspect of these liquids was studied separately, and we will present the results of this study in a separate manuscript. Figure 4a shows that the aluminum in three the ILAs undergoes deposition at −0.01 V, continuing until the vertex potential is close to −0.1 V. In the acidic chloroaluminate, the active species that are responsible for Al deposition and dissolution can be attributed to Al_2_Cl_7_^–^ or AlCl_2_(L)*_n_*^+^. A representation of the mechanism of the reaction of Al_2_Cl_7_^–^ is as follows
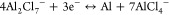
In the anodic reaction, Al_2_Cl_7_^–^ is produced when AlCl_4_^–^ reacts with the aluminum from the anode, giving the reaction
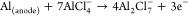
In the cathodic reaction, the Al_2_Cl_7_^–^ diffuses to the electrode and is discharged to allow aluminum deposition as shown^31^
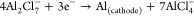


Some studies reported that in amide systems such as guanidine,^32^ urea,^9,12^ and acetamide,^21,33^ besides AlCl_4_^–^ and Al_2_Cl_7_^–^, other species, for instance, AlCl_2_·(amide)*_n_*^+^ (*n* = 1, 2) and neutral AlCl_3_·(amide)*n*, were obtained and were considered as contributing to reversible Al electrodeposition/dissolution as shown below. Here, the potential corresponding to the onset of anodic degradation was determined by the intercept of the tangents to the two parts of the voltammetric curve in this region
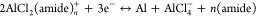


However, in the neutral composition AlCl_3_:Amide (1.0), there is no significant electrochemical activity due to the absence of Al_2_Cl_7_^–^, and so it can be considered that AlCl_2_·(amide)*_n_*^+^ cannot be reduced alone. The proposed reaction can be seen below:^34^
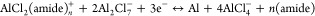


The voltammogram in Figure 4a also shows that the current densities generated from the three ILAs are significantly different. The current densities observed were around 79.62, 58.82, and 11.54 mA cm^–2^ for AlCl_3_:Guanidine, AlCl_3_:Acetamidine, and AlCl_3_:Urea, respectively, for the anodic peak. The lower peak of the Al dissolution current, 15.54 mA cm^–2^, was recorded using an electrolyte of AlCl_3_:Urea while the higher one from AlCl_3_:Guanidine is 110.28 mA cm^–2^. The large difference in current densities is a consequence of ion mobility and possibly electron-transfer effects. It has been demonstrated that the higher current peak of AlCl_3_:Guanidine is correlated to a higher conductivity and lower viscosity. This is not a bulk concentration effect as the total Al ion concentration is the highest in the urea liquid (see an earlier discussion), where the lowest current density is observed. On the other hand, it cannot be excluded that the different ionic speciation influences the dissolution deposition kinetics.

The LSV data presented in Figure 4b show that the onset of anodic degradation for the three liquids, AlCl_3_:Urea, AlCl_3_:Guanidine, and AlCl_3_:Actemidine, occurs at +2.12, +2.30, and +2.33 V, respectively, vs. an Al^(III)^/Al reference^a^. This phenomenon could be attributed to the oxidation process of AlCl_4_^–^ or Lewis bases (guanidine, acetamidine, or urea) adduct species. Hence, the urea liquid has the lowest resistance to anodic degradation, with the other two electrolytes performing slightly better. This is significant because lower anodic stability will limit the cycle life of an operational battery device.

### 3.4 Symmetrical Coin-Cell Tests

**Figure 5:**
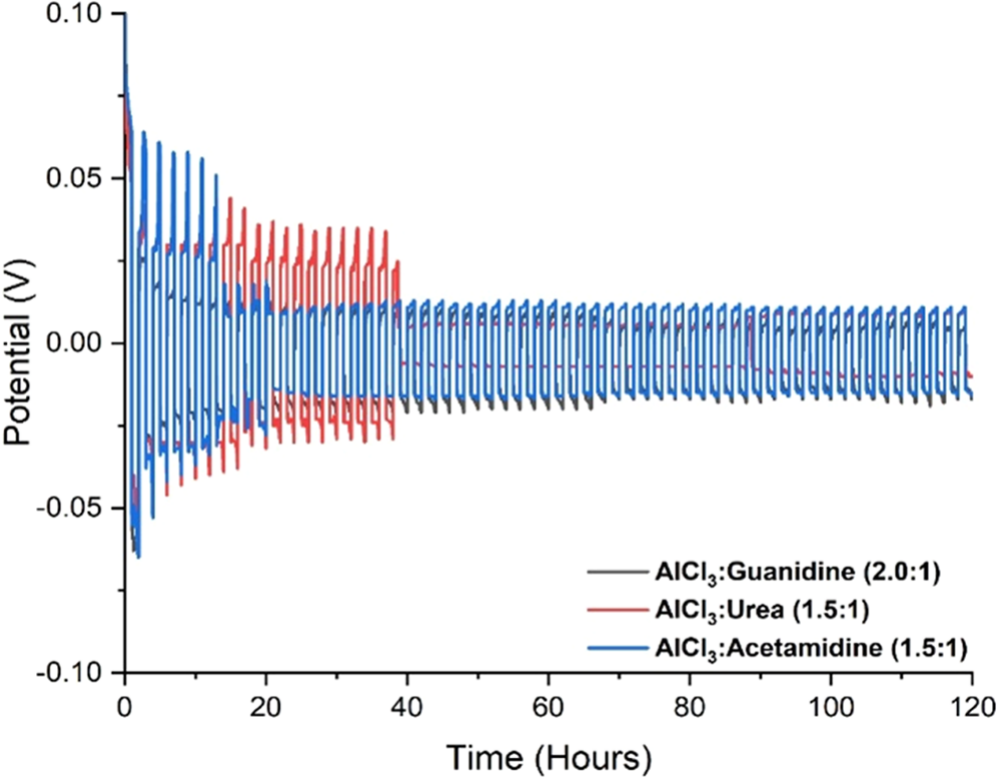
Symmetrical coin-cell testing using the Al foil anode and cathode and ILA electrolyte at a current density of 0.1 mA cm^–2^ with a time limit of 1 h while charging and discharging for up to 120 cycles.

To assess the long-term stability of the interface between Al metal and the electrolyte ILA under dynamic conditions, galvanostatic cycling tests were conducted on symmetrical Al/Al cells using the three investigated electrolytes. Figure 5 shows a representative voltage profile of a symmetrical cell during Al dissolution/plating at a current density of 0.1 mA cm^–2^ with a time limit of 1 h while charging and discharging for up to 120 cycles. For each liquid, the appearance of the potential profile is typical for this type of symmetrical cell, showing both deposition and dissolution of Al in the cathodic and anodic cycles, respectively. During each individual cycle, the shapes of the potential profiles are quite consistent, indicating that neither anode passivation nor cathode fouling occurs even after long periods of cycling. Initial results showed large overpotentials for symmetric cells, but these overpotentials gradually decreased and stabilized over time. This initial high overpotential is probably due to the presence of the aluminum oxide (Al_2_O_3_)-passivating layer or hydrogen evolution reaction from the residual/coordinated water.^35,36^ During the initial aluminum deposition step, symmetrical cell testing for AlCl_3_:Urea recorded the maximum overpotential at around 66 mV, decreasing to 25 mV after 19 cycles and subsequently remaining stable at 10 mV. For AlCl_3_:Acetamidine, the overpotential was 64 mV and then decreased to 18 mV during 10 cycles and remained stable at 11 mV. Interestingly, for AlCl_3_:Guanidine, the overpotential was shown in one cycle at 62 mV and remained stable up to the end at 6 mV. Hence, the performance of the AlCl_3_:Urea ILA was the poorest in these tests and the stabilization of the Al/ILA interface was much faster with the AlCl_3_:Guanidine electrolyte.

### 3.5 Aluminum|Pyrolytic Graphite Coin-Cell Tests

**Figure 6:**
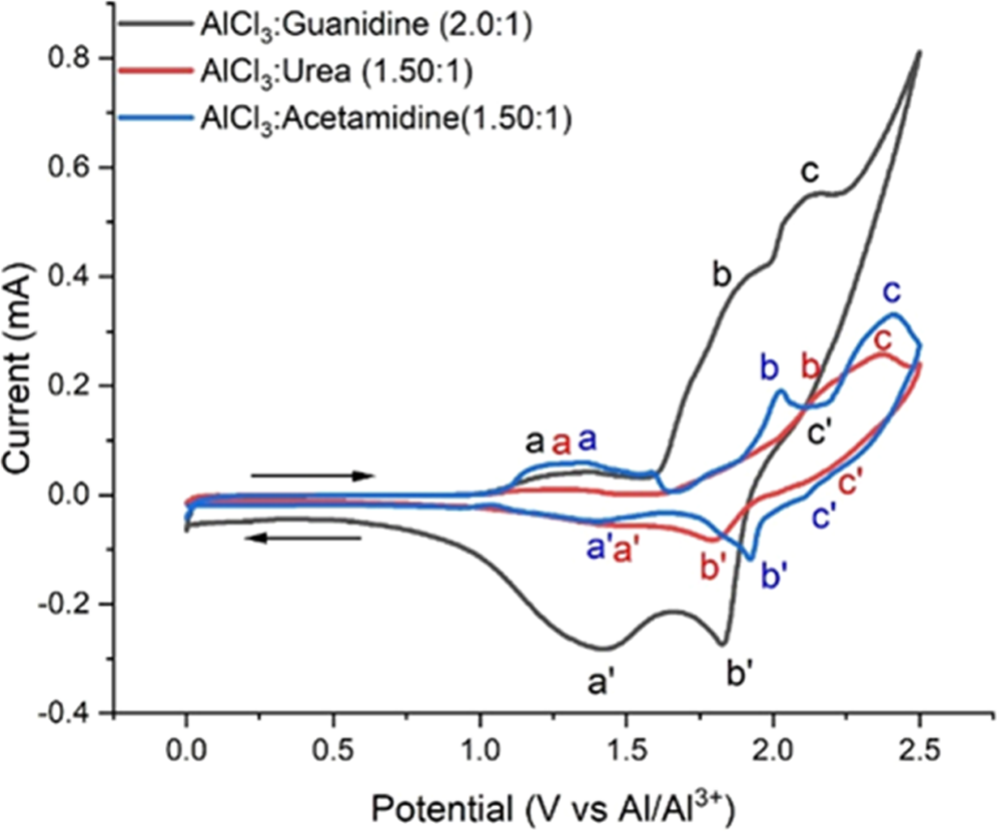
Cyclic voltammetry (CV) of the three ILAs using a PG working electrode at a scan rate of 0.1 mV s^–1^ (against an Al^(III)^/Al reference electrode). Oxidative intercalation processes are indicated as peaks *a*, *b*, and *c*. Corresponding reductive deintercalation processes are indicated as peaks *a*′, *b*′, and *c*′.

To further explore the performance of each ILA electrolyte, coin cells were subsequently assembled using an Al foil anode in combination with a PG cathode material. Cyclic voltammograms (CVs) of the ILA electrolytes using the pyrolytic graphite (PG) working electrode of the coin cell and an Al foil as a counter electrode are presented in Figure 6. The initial comparison of the CV responses for the three liquids reveals significant differences in the maximum current density of each CV. The peak currents of the guanidine electrolyte are the largest. This may be due to the observed differences in conductivity and viscosity, as discussed previously. However, while the PG electrode was fabricated from an identical mass of PG in each case, we have exerted no control of the surface area of the PG between electrode samples.

According to previous studies,^1,36^ the anion intercalation of the chloroaluminate into graphite has been proposed, and it can be generally expressed as a reversible reaction with the anode and cathode components as follows:
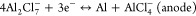

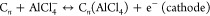
where *n* is the molar stoichiometry of carbon atoms representing an oxidative process of the graphite. Figure 6 shows that there is a similar overall CV pattern for the three ILAs, indicating similar graphite intercalation processes. There are three oxidation peaks, peaks *a*, *b*, and *c*, assigned as intercalation processes, and three corresponding reduction peaks, peaks *a*′, *b*′, and *c*′, assigned as deintercalation processes. According to Sun et al.,^37^ the polarization could be affected by either the size of the ionic diameter and coordination of the aluminum ions restricting the intercalation/deintercalation process or the passivation film on the anode side of the aluminum foil preventing the deposition and dissolution of aluminum. In addition, the redox potentials (*a*, *b*, *c*) at which these processes occur may reflect the energetics, population, and distribution of intercalation sites. Similar sequential intercalation processes are observed for Li-ion systems where the energy (potential) for each process is related to the population and distribution of the Li atoms in the graphite matrix.

**Figure 7:**
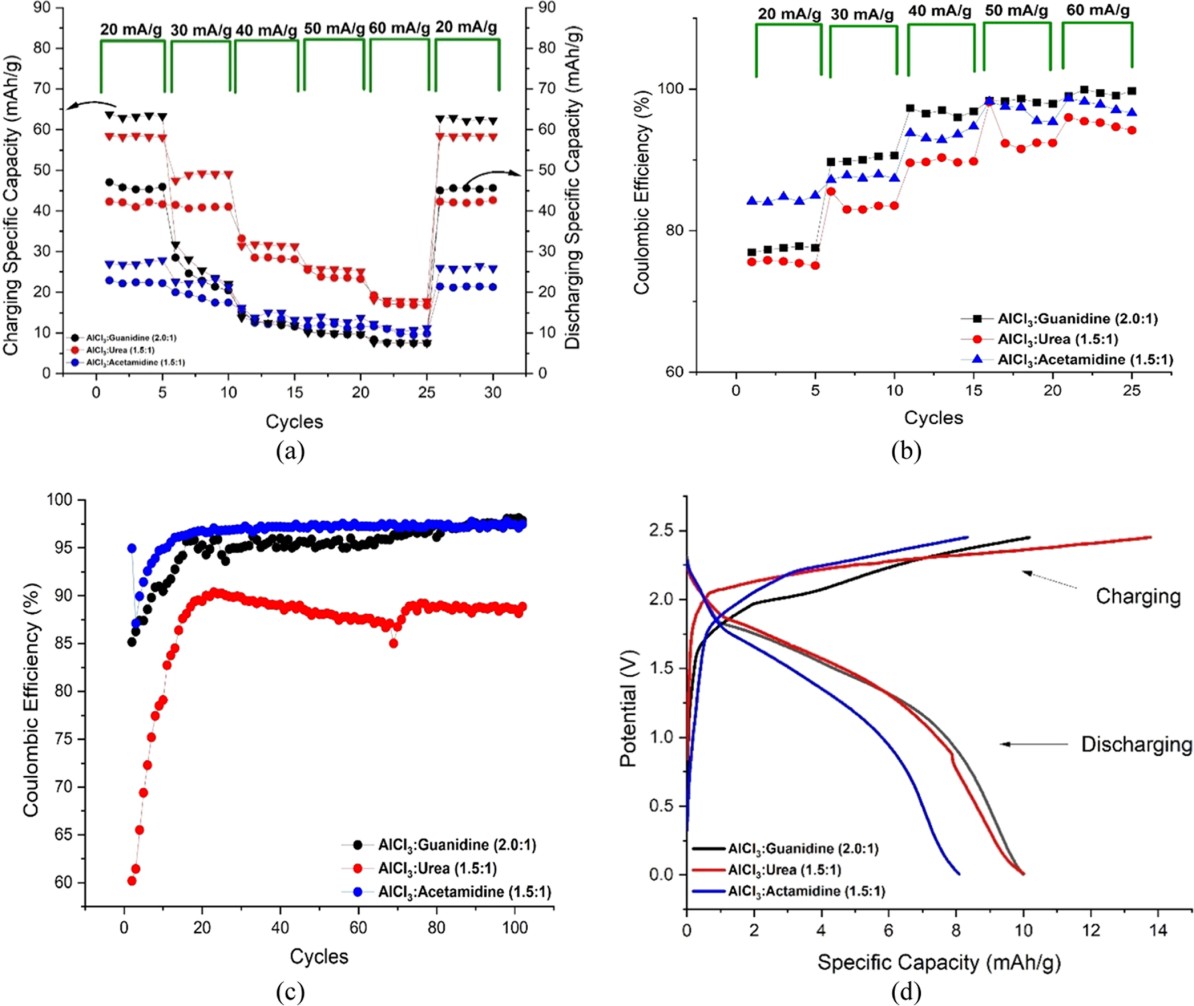
Performance data for coin cells fabricated from an Al foil anode, PG cathode, and ILA electrolyte; (a) charging (▼)/discharging (●) specific capacity as a function of the applied current; (b) Coulombic efficiency (CE) profile as a function of the applied current; (c) Coulombic efficiency for 100 cycles at 60 mA g^–-1^; and (d) charging/discharging potential profiles at the 100th cycle (cells from part (c)).

The bulk charge/discharge characteristics of the coin cells were subsequently explored. The data presented in Figure 7a show the charging and discharging capacities (mAhr g^–1^) over five successive cycles for the cells fabricated using the three ILA electrolytes. The charge/discharge cycles were repeated using experimentally applied current densities of 20, 30, 40, 50, and 60 mA g^–1^ with an experimental cutoff voltage between +0.01 V (during the discharge cycle) and +2.45 V (during the charge cycle). The total cell capacity (maximum theoretical) was defined by the mass of the PG cathode, which was fixed at 13 mg here. In battery terminology, the C-rate describes the rate at which a battery is charged or discharged in relation to its capacity (mAhr) over a period of 1 h. Hence, a rate of 1C requires sufficient current to fully charge or discharge the cell in 1 h. For a variety of reasons, including mass transport and electron-transfer kinetics, the measured capacity of a cell can be dependent on the C-rate. Here, the maximum theoretical specific capacity of the graphite (PG) cathode (as defined by the manufacturer) is around 372 mAhr g^–1^.^38^ For a cathode mass of 13 mg, this yields a cell capacity of 4.836 mAhr and so a 1C charge/discharge current of 4.836 mA. The applied currents (0.26 0.39, 0.52, 0.64, and 0.77 mA; Figure 7a) in our experiments correspond to C-rates of 0.05C, 0.08C, 0.11C, 0.13C, and 0.16C, respectively. These data are summarized in Table 3. These values of the discharge C-rate were chosen as representative of small-scale applications for general battery use and therefore represent a measure not only of performance but also stability.

**Table 3:** Charge/Discharge Current Densities, Equivalent Cell Currents, and C-Rates for Al|PG Coin Cells for Which the Results are Presented in Figure 7 a^38^

charge/discharge current density (mAhr g^–1^)	absolute cell current (mA)	equivalent C-rate
20	0.26	0.05
30	0.39	0.08
40	0.52	0.11
50	0.65	0.13
60	0.78	0.16

a These are based on the mass of the PG cathode (13 mg) having a theoretical capacity of 372 mAhr g^–1^.^38^

Although the total capacities of these cells are relatively low (compared with the maximum theoretical capacity), we attribute this to the fact that the viscosities of all liquids here may preclude them from accessing the microporous surface areas of the PG cathode material. Nevertheless, the data in Figure 7a show several interesting features. First, for all of the ILA cells, the values of capacity in the charge and discharge measurements were consistent between successive cycles over the course of these experiments. This is reassuring in the sense that the cells were well fabricated, airtight, and stable. Second, the highest capacity determined for these cells was consistently achieved by the guanidine electrolyte, followed by the urea ILA. It is clear that as the charge/discharge current density, and so C-rate, is increased during successive cycles (1 to 25) from 20 mA g^–1^ (0.05C) to 60 mA g^–1^(0.16C), the measured capacity of the cells drops dramatically. This is not uncommon with such cells and can probably be attributed to polarization caused by slow mass transport. However, in cycles 26–30, Figure 7a shows that when the measurements are repeated at a low current density and C-rate (20 mA g^–1^ (0.05C)), the capacities are restored to their former values. This is good evidence that no irreversible chemical damage or degradation has been caused by cycling the cells at a high C-rate.

At the initial current (20 mA g^–1^), AlCl_3_:Guanidine shows higher charging/discharging specific capacity compared to the two other systems (AlCl_3_:Urea and AlCl_3_:Acetamidine). The average of five cycles of the charging specific capacity for the three systems are 63, 58, and 27 mA g^–1^, respectively, and 46, 41, and 23 mAhr g^–1^ for the discharging specific capacity, respectively. The charging/discharging capacity in this study is relatively low compared to that in other studies, but the results are almost the same as in the study conducted by Bogolowski et al.,^13^ who used a pyrolytic graphite produced by Panasonic as a cathode. The Coulombic efficiency (CE) for these cells as a function of the charge rate is presented in Figure 7b. It can be seen that the higher the applied current, the higher the Coulombic efficiency. The average CE values obtained over five cycles were 77.44, 84.42, and 75.51% at a current density of 20 mA g^–1^ and 98.37, 97.67, and 90.10% at 60 mA g^–1^ for AlCl_3_:Guanidine, AlCl_3_:Acetamidine, and AlCl_3_:Urea, respectively.

In a subsequent experiment, three Al|PG cells were tested under the same conditions for 100 cycles at a higher current density of 60 mA g^–1^ (0.16C) in order to evaluate the longer-term stability and CE^b^. These data are shown in Figure 7c. All three cells showed a conditioning period of ca. 20 cycles, after which a stable CE efficiency was achieved. The urea cell performed quite poorly here, showing a consistently low CE as well as significant fading and variability in the efficiency at this rate. Both acetamidine and guanidine electrolytes performed much better, having a stable CE after the first conditioning period. It was found that after 100 cycles, AlCl_3_:Guanidine shows the highest CE of 98.12%, followed by AlCl_3_:Acetamidine (97.10%) and AlCl_3_:Urea (88.17%). This lower CE of the AlCl_3_:Urea system may be attributed to the relatively poor resistance toward the oxidation process (as shown in Figure 4b) and also the lower conductivity (higher viscosity) (Table 1).

During the above experiment (Figure 7c), the potential profiles for charge and discharge were also collected. The cell potential versus specific capacity profiles for the cells at the 100th cycle are shown in Figure 7d. The charging profiles of all three electrolytes are quite similar in performance; however, the urea system takes more charge before the cutoff voltage is reached. This may be due to the lower anodic stability discussed previously. Importantly, these profiles show that during discharge, the cell voltage of the guanidine electrolyte cell closely matches that of the urea-based system. The voltage of the acetamidine ILA cell drops more quickly with the capacity at this discharge rate.

Overall, in all of the cell tests, the AlCl_3_:Guanidine electrolyte consistently performs better than the AlCl_3_:Urea electrolyte, where the latter has been considered as a strong candidate for rechargeable Al-based batteries.

## 4 Conclusions

In this paper, we present the outcome of an academic study into the properties of some new and relatively novel chloroaluminate ionic liquid analogues. We present not only the liquid-phase rheological and electrochemical properties but also correlate these with the prototype battery devices in which their application is suited and may make significant technological contributions. A comparison of the standard AlCl_3_:Urea electrolyte with AlCl_3_:Guanidne and AlCl_3_:Acetamidine electrolytes was conducted. Comparatively, both AlCl_3_:Guanidine and AlCl_3_:Acetamidine electrolytes demonstrated superior rheological characteristics (conductivity, viscosity, and activation energy) and served as better media for Al dissolution and deposition than the AlCl_3_:Urea system. In addition, both the guanidine and acetamidine electrolytes showed better stability in the anodic region, which is beneficial for battery performance. In coin-cell tests, we observed that due to high conductivity, low viscosity, and low activation energy, both electrolytes have slightly higher Coulombic efficiencies (98.12% for AlCl_3_:Guanidine; 97.10% for AlCl_3_:Acetamidine) than that of the AlCl_3_:Urea system (88.17%). In addition, symmetrical cell testing revealed that the amidine electrolytes have lower overpotentials for the deposition and dissolution processes. Lastly, as the two ILAs based on guanidine hydrochloride and acetamidine hydrochloride exhibit superior battery performance and are also low in price, they are considered to be valuable alternatives to ABB systems, especially for industrial scale-up production.

## Supporting Information Available

Additional data showing the appearance of the liquids and their response to repetitive voltammetric cycling, including ILAs of AlCl_3_:Guanidine, ACl_3_:Actemidine, and AlCl_3_:Urea at room temperature and CV of five cycles of AlCl_3_:Guanidine, ACl_3_:Actemidine, and AlCl_3_:Urea liquids at a scan rate of 10 mV s^–1^ at room temperature (PDF)

## Footnotes

a Here, the potential corresponding to the onset of anodic degradation was determined by the intercept of the tangents to the current baseline of the voltammogram and the increasing anodic current toward the potential limit.

b A maximum number of 100 cycles was chosen for this study because of the time scales imposed by the relatively low C-rate. Measurements of up to 1000 cycles and beyond would be necessary to establish longer-term stability.
