# Supplementary material for: Comparative Study of Guanidine-, Acetamidine- and Urea-Based Chloroaluminate Electrolytes for an Aluminum Battery
Source: J Phys Chem C Nanomater Interfaces. 2023 Sep 15;127(38):18891–901. doi: 10.1021/acs.jpcc.3c05287 (PMC10544989; doi:10.1021/acs.jpcc.3c05287)
Supplement: Supplementary file 1 — jp3c05287_si_001.pdf [file jp3c05287_si_001.pdf]

Electronic Supporting Information (ESI)

A Comparative Study of Guanidine-, Acetamidine- and Urea-based Chloroaluminate Electrolytes for an Aluminium Battery

Iwan Sumarlan,[a,b] Anand Kunverji [b], Anthony J. Lucio [b] , A. Robert Hillman[b] , and Karl S. Ryder [b*]

a Department of Chemistry, University of Mataram, Jl. Majapahit. No. 62, Mataram, Lombok, Indonesia

b Centre for Sustainable Materials Processing, School of Chemistry, University of Leicester, Leicester, LE1 7RH, U.K.

* Corresponding author:

Figure S1. Ionic Liquid Analogues (ILAs) of $AlCl_3$:Guanidine, ACl_3:Actemidine and $AlCl_3$:Urea at room temprature

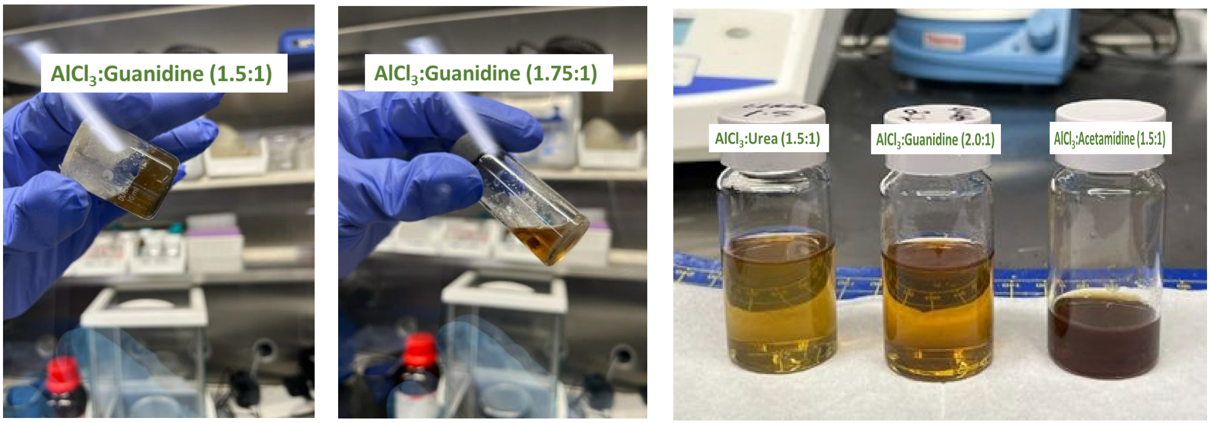

Figure S2. Cyclic Voltammetry (CV) of five cycles of (a) $AlCl_3$:Guanidine, (b) ACl_3:Actemidine and (c) $AlCl_3$:Urea liquids with scan rate of 10 mV s^{-1} at room temprature.

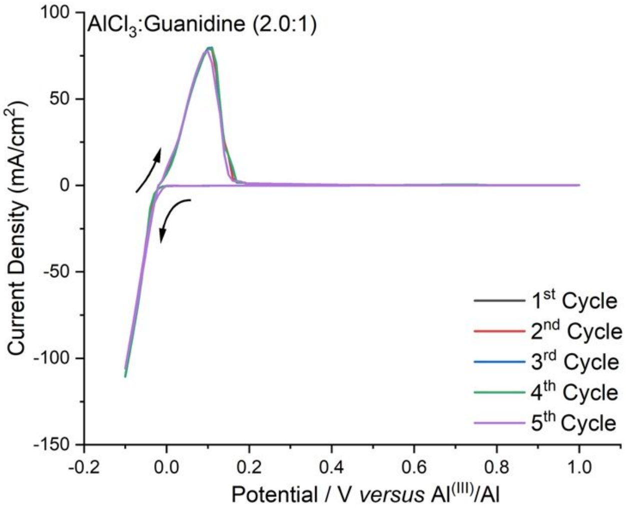

(a)

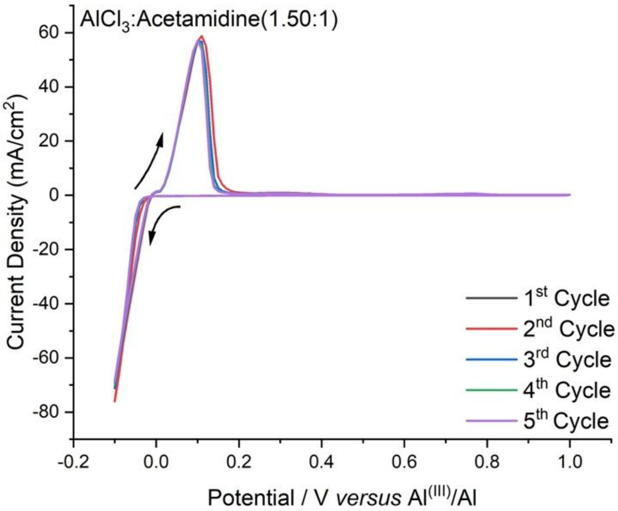

(b)

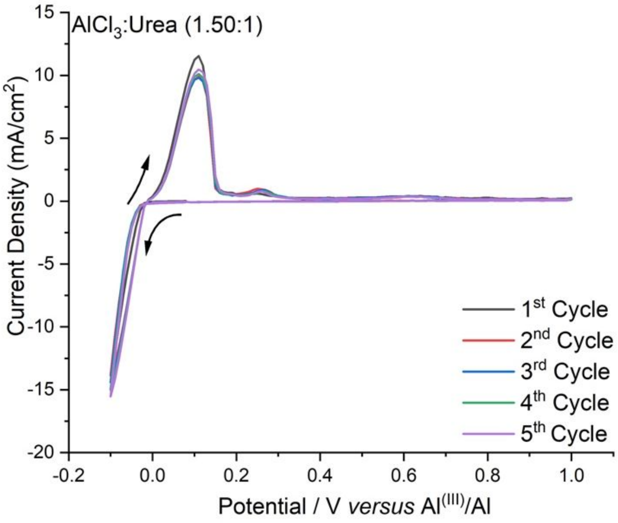

(c)
